# International roadmap for artificial gravity research

**DOI:** 10.1038/s41526-017-0034-8

**Published:** 2017-11-24

**Authors:** Gilles Clément

**Affiliations:** KBRwyle, 2400 NASA Parkway, Houston, TX 77030 USA

## Abstract

In this paper, we summarize the current and future research activities that will determine the requirements for implementing artificial gravity (AG) to mitigate the effects of long duration exposure to microgravity on board exploration class space vehicles. NASA and its international partners have developed an AG roadmap that contains a common set of goals, objectives, and milestones. This roadmap includes both ground-based and space-based projects, and involves human subjects as well as animal and cell models. It provides a framework that facilitates opportunities for collaboration using the full range of AG facilities that are available worldwide, and a forum for space physiologists, crew surgeons, astronauts, vehicle designers, and mission planners to review, evaluate, and discuss the issues of incorporating AG technologies into the vehicle design.

## Introduction

In the past, countermeasures to mitigate the physiological effects of deconditioning due to microgravity were delivered in a piece-meal fashion, e.g., fluid loading to counteract effects on the cardiovascular system, and exercise to mitigate muscle and bone loss. Although these countermeasures have greatly reduced health risks due to physiological deconditioning, they involve extensive crew time and a great deal of equipment. Since artificial gravity (AG) can reproduce Earth-like gravity, it could be used to simultaneously mitigate the effects of microgravity on all the physiological systems. AG can be generated by continuously rotating the entire spacecraft, or part of the spacecraft, or by means of an onboard short-radius centrifuge that the crewmembers can use intermittently. The parameters that are most effective for mitigating physiological deconditioning in microgravity (rotation rate, radius of the centrifuge, and duration and frequency of AG exposure) need to be determined early in the exploration mission planning process to inform optimal decisions on the vehicle capabilities.^1^ In addition, potential side effects of intermittent or continuous rotation need to be understood and addressed. These side effects, which include motion sickness, disorientation, and falls, are caused by the Coriolis and cross-coupled angular accelerations generated by head and body motion in a rotating environment. Apathy, fatigue, and impairment in cognitive performance have also been observed in volunteers living in slowly rotating rooms;^2,3^ therefore, AG research requires an integrative approach that includes physiological, behavioral, and human factor aspects.

**Fig. 1:**
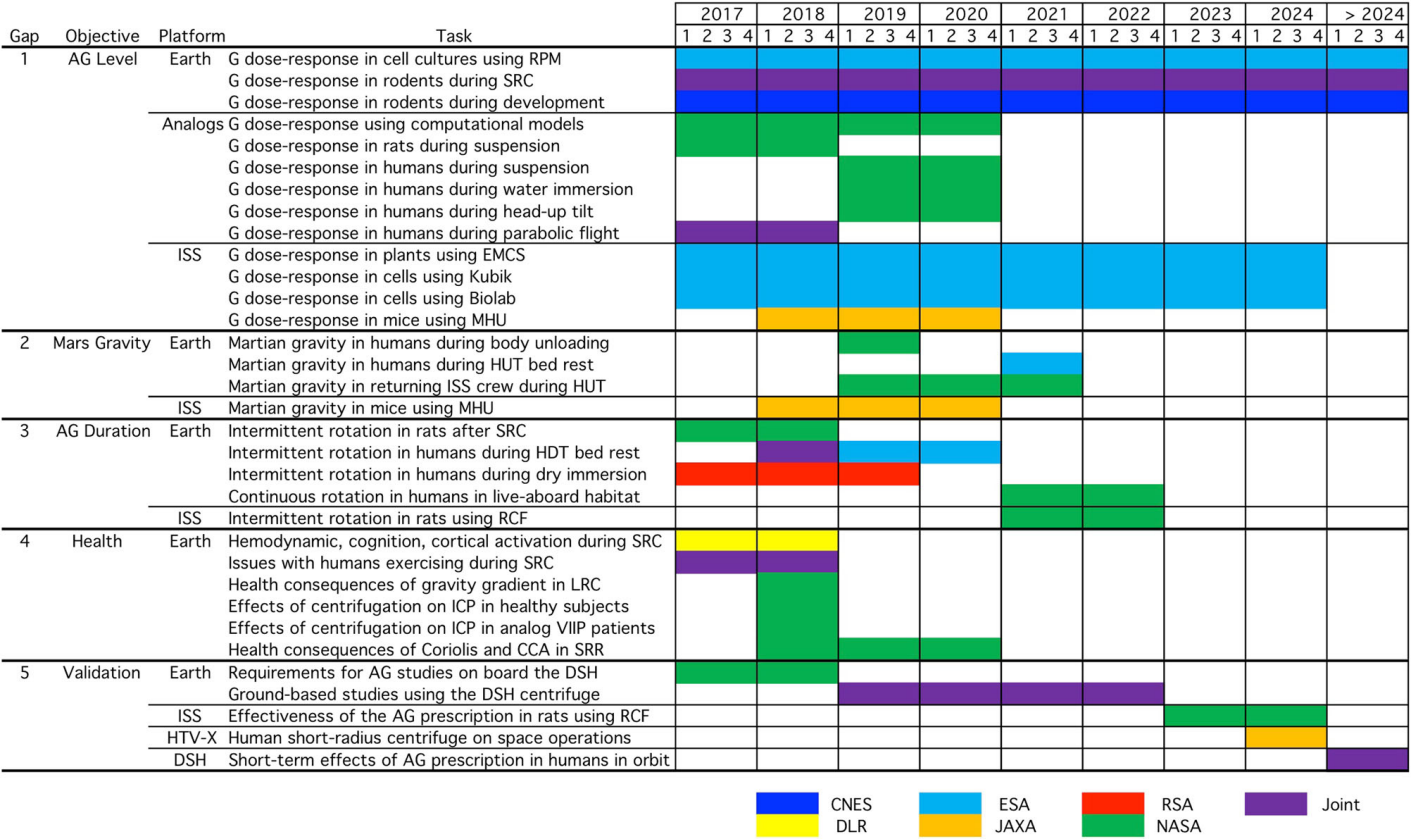
International AG roadmap. The international AG roadmap lists the research activities (tasks) that address each of the five identified knowledge gaps. Research projects are ground-based (Earth, Analogs) or space-based (ISS, DSH, HTV-X). Projects are planned on board the ISS up to 2024 and other vehicles/habitats thereafter. CCA cross-coupled angular accelerations, EMCS European Multi Cultivation System, HUT head up tilt, HDT head down tilt, ICP intracranial pressure, LRC large radius centrifuge, MHU Mouse Habitat Unit, RCF Rodent Centrifuge Facility, SRC short-radius centrifuge, SRR slow rotating room, RPM random positioning machine, VIIP visual Impairment due to Intracranial Pressure

Humans have had limited exposure to AG (see review in ref. 4) and no AG capability exists for humans on board the International Space Station (ISS). A complete research program is warranted to determine both the requirements and constraints of intermittent and continuous rotation of humans in space before deciding whether AG should be implemented during a Mars mission. Until recently, however, no coordinated research plan existed. The development of an international roadmap for AG research was recommended during a workshop on “Research and Operational Considerations for Artificial Gravity Countermeasures” what was held at NASA Ames Research Center in February 2014.^5^ Roadmaps effectively translate abstract needs and concepts into concrete research activities that specify deliverables and the resources necessary to make progress in a timely fashion. A coordinated AG roadmap will provide information for the space vehicle designers, mission planners, and managers regarding AG requirements for a manned mission to Mars. It will also provide a framework that facilitates collaboration using the full range of available AG facilities worldwide. To this end, NASA organized a workshop in February 2016 in Galveston, Texas, and invited representatives from NASA and from space agencies of France, Germany, Europe and Japan, as well as scientists who were actively involved in AG research. This paper is a review of the roadmap that was discussed during this workshop (Fig. 1).

## Organization of this review

The overarching goal of AG research is to inform managers and mission designers of specific requirements and the costs and benefits of AG for any given mission scenario. AG can be adjusted by varying the rotation rate of the spacecraft or centrifuge or varying the distance of the habitat or crewmember relative to the axis of rotation. These AG parameters impact vehicle design and operations. The questions that need answers are (a) what evidence is there to support the requirement for AG during a long-duration mission; (b) what design parameters should be levied on the engineers; and (c) what prescriptions (gravity level, duration, frequency) should be recommended to the crewmembers? In addition, recommendations must also be provided regarding additional, complementary countermeasures that will ensure the health and performance of crewmembers who participate in long-duration missions. These questions must be answered and recommendations must be provided before the design of the spacecraft and mission is completed.

The international roadmap for AG research uses the same management architecture as other projects in NASA’s Human Research Program.^6^ The architecture is based on (a) evidence that forms the basis of the existence of a risk to the human health, (b) gaps in current knowledge on how to characterize or mitigate the risk, and (c) tasks that produce the deliverables needed to close the gaps and reduce the risk. AG is considered a countermeasure that might include some intrinsic risk. Five gaps in our knowledge of how to implement AG in a space vehicle were identified: the minimum AG level (Gap 1) and duration (Gap 2) required to mitigate the effects of microgravity; the potential effects of Mars gravity (Gap 3); the health consequences of Coriolis, cross-coupled acceleration, and gravity gradient (Gap 4); and whether the AG prescription determined during ground-based studies in humans will be effective, acceptable, and safe for the crew in space (Gap 5).

## Gap 1—AG level

We must understand how gravity affects fundamental physiological processes before we can understand physiological adaptation during spaceflight and develop the most efficient countermeasures. The first step is to define the relationship between gravitational dose and physiological response by assessing gravity levels ranging from 0 to 1 g. The second step is to identify the range of gravity level in which physiological response is the closest to normal, i.e., response to Earth gravity. This analysis will determine the operating range of AG levels that is most likely to be effective as a countermeasure. Although gravitational dose–response curves have been obtained for some biochemical systems in animals,^7^ these dose–responses are unknown for most human physiological systems.

It is important to note that, in addition to the aims outlined above, these studies will document and improve our understanding of the mechanisms of adaptation to chronically altered gravity levels, including fractional gravity such as on the Moon and Mars, and hypergravity.

### Studies in cell and animal models

Several investigators have proposed using the random positioning machine (RPM) to study the effects of a range of gravity levels on cell cultures.^8–12^ A RPM rotates biological samples along two independent axes; this changes the orientation and eliminates the effect of gravity on the samples. Theoretically, two methods can be used to simulate a range of partial gravity levels using the RPM: rotating for longer or faster in one specific direction than for other directions, or stopping the rotation for short periods when the gravity vector is pointing downwards. However, each of these methods seems to give different results. Also, it is not possible to use the RPM in space. Without a direct comparison between ground and space data, it is difficult to conclude whether biological reactions and organismic responses are caused by the conditions of simulated partial gravity or by any of the possible side effects of the simulation technique.^13^

**Fig. 2:**
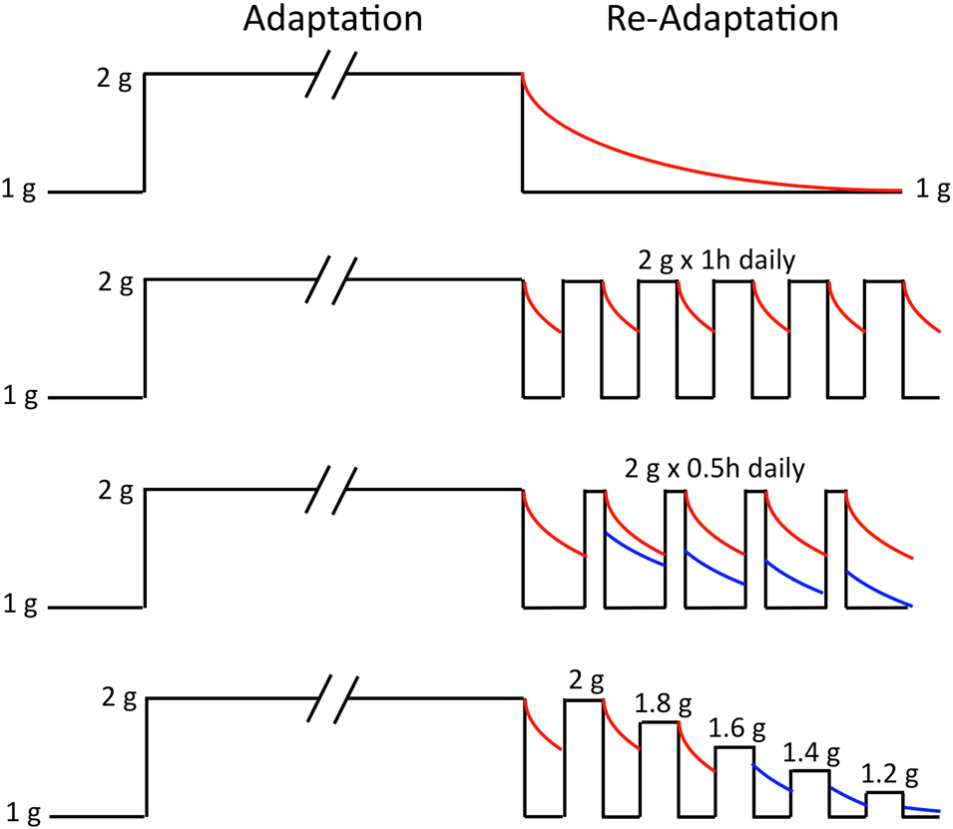
Design of ground-based experiments for investigating the threshold in centrifugation force level and duration. Animals are exposed to continuous rotation at 2 g for several weeks (Adaptation). Centrifugation stops and re-adaptation of the physiological responses to Earth gravity is then compared for various intermittent periods (1 h daily, 0.5 h daily) or intervals. Once the minimum duration of centrifugation force preventing re-adaptation to 1 g has been identified, other animals are submitted to various levels of centrifugation force (1.8–1.2 g) to determine the minimum level that prevents re-adaptation to 1 g. Red curves show no retention of adaptation; blue curves show retention of adaptation. (Adapted from ref. 16)

The results of hypergravity studies on Earth can potentially shed some light on the effects of partial gravity in space because data demonstrate a remarkable continuum of response across the hypogravity and hypergravity environments.^14,15^ For example, centrifugation on Earth can be used to study the re-adaptation thresholds for the level and duration of centrifugal force (Fig. 2). In these protocols, samples are exposed to hypergravity (e.g., 2 g) for several weeks; centrifugation is stopped, and after various durations it is re-instated at different gravity levels. The minimum duration and level of centrifugal force required to prevent re-adaptation of various physiological responses to 1 g can be extrapolated to partial gravity using this method.^16^

Centrifuges that are available on the ISS for studying the effects of partial gravity on biological processes in plants, cells, and animals include European Space Agency's (ESA) European Modular Cultivation System (EMCS), Kubik, and Biolab, and JAXA’s Mouse Habitat Unit. EMCS is dedicated to experiments on plants, including studies of gravity threshold on early development and growth, and can provide AG levels as low as 0.001 g. The first studies on the effects of fractional gravity on board the ISS were performed on plants, and the results showed that gravity sensing was saturated between 0.1 g and 0.3 g.^17,18^ Kubik is a small incubator in which small containers of biological samples can be exposed to AG levels from 0.2 g to 2 g in 0.1 g increments. Biolab supports biological research on small plants, small invertebrates, microorganisms, animal cells, and tissue cultures. It includes an incubator equipped with centrifuges that can generate AG levels from 0.01 to 2 g.^19^

Animal models of simulated microgravity, such as tail suspension in rats, have yielded important information on how the cardiovascular, neuromuscular, and neuroendocrine systems adapt to microgravity. The rat is preferred for rodent studies because rats have a larger mass than mice, making them more sensitive to the effects of partial gravity. However, the rodent centrifuge on the ISS can only accommodate mice. The centrifuge in the JAXA Mouse Habitat Unit can spin six cages, each containing an individual mouse, at a distance of 15 cm from the axis of rotation, generating AG levels ranging from 0.1 to 4 g for up to 6 months.^20^ Studies have shown that the mouse is a valuable model for studying musculoskeletal and cardiovascular changes during microgravity.^21^ Also, because the mouse genome has been extensively studied, physiological mechanisms can be examined on a gene-specific basis. In-flight centrifugation of mice will provide valuable data on how mammalian health and behavior is affected by partial gravity. Research will focus on bone loss, muscle atrophy, changes in intracranial pressure, vascular flow, aerobic capacity, immunity, and inner ear function. Research using mice will also provide information indicating whether the partial gravity of the Moon or Mars sufficiently protects against the physiological changes that occur in 0 g, or whether astronauts will require additional countermeasures while on these planets.

### Studies in humans

The AG roadmap outlines several approaches for studying gravity in human subjects. Parabolic flight will be used to characterize the relationship between gravitational dose and acute responses of the cardiovascular, cerebrovascular, ocular, muscular, and sensorimotor systems. Previous parabolic flight studies were performed in the U.S., Canada, and Europe during short, repeated exposures to 0.16 and 0.38 g.^22^ ESA and its partners from the International Life Science Working Group are coordinating a partial gravity parabolic flight campaign in spring 2018. During this campaign an integrated study of a single test subject during several flights and using several experimental protocols will measure the responses of multiple systems at 0.25, 0.5, and 0.75 g.

The effect of graded head-out water immersion will be investigated using subjects seated in an upright posture while they are immersed up to the hip, heart, or neck. The hydrostatic water pressure on the body counteracts the intravascular hydrostatic pressure gradients, simulating what is expected to occur in Martian gravity, lunar gravity, or microgravity.^23,24^

Partial gravity will be simulated by placing subjects supine with their heads tilted upward 11.5° to 30° from horizontal at increments of ~6°, thus simulating gravity at increments of 0.1 g from 0.2 g–0.5 g along Gz axis the subject’s body. Subjects will sleep in the horizontal position. Five days of bed rest in a head down tilt induces orthostatic intolerance, endocrine response changes, and changes in muscle and bone markers, similar to those in actual spaceflight.^25–27^ Bed rest of more than 5 days will be studied to determine effects on sensorimotor function.

Suspension techniques can be used to simulate partial gravity on subjects while they perform locomotion studies and training exercises. Overhead suspension systems typically use cables, springs, and air bearing rails to partially or fully unload the subject’s weight. NASA’s partial gravity simulator, also known as POGO, is used to train astronauts and evaluate their ability to perform tasks in simulated partial gravity and microgravity.^28^ Massachusetts Institute of Technology’s partial gravity simulator, known as Moonwalker, also uses a spring-offset system to study body movements in simulated partial gravity as low as 0.05 g. The body can be suspended for as long as necessary using these systems, but the subject’s degree of freedom is limited.^29^

Lower body positive pressure treadmills can be used to study muscle activation and gait patterns during body weight unloading. An inflated air chamber around the lower body lifts the subject upwards at the hips, effectively reducing gravitational forces at the feet, and reducing the apparent weight of the body up to 80%.^30^

Ballasted partial gravity systems have been used to study human operational activities in an underwater environment. The subject wears a body harness with attached weights that can be adjusted to provide the correct buoyancy. This type of simulation is best suited for quasi-stationary studies (such as load lifting, ingress/egress) that minimize the effect of water drag on the subject’s movement. However, recent studies using a ballasting system and an underwater treadmill have provided valuable information on the dynamics of human gaits in partial gravity.^31^

Computational models can predict how physiological response might adapt to different gravity levels and will provide data that are unavailable from experimental-based analog studies. Computational modeling can also simulate the effect of long duration exposures to partial gravity that might be impossible or too costly to investigate in analogs. Additionally, models will be used for hypothesis testing and parametric studies to help refine the experimental protocols for ground or flight studies. The NASA Digital Astronaut Project is currently working with bone specialists to establish models of bone loss due to skeletal unloading, models of renal stone formation, and changes in heart shape and stress distribution in microgravity.^32^ Once validated using spaceflight and bed rest data, these models will be used for predicting changes in these physiological systems during partial gravity.

## Gap 2—Mars gravity level

The studies outlined in gap 2 of the AG roadmap will quantify the effects of Martian gravity (0.38 g) using centrifugation of cell cultures and animals on board the ISS. Recent studies of mice what were exposed to partial weight-bearing suspension for 21 days have shown that Martian gravity, as simulated on Earth, is not sufficient to protect against the bone loss observed in microgravity, but it does mitigate reduction in soleus mass.^33,34^ The JAXA ISS mice centrifuge will be used to investigate if the same effects are seen during simulated Martian gravity in orbit, and these studies will help determine whether astronauts will require countermeasures to mitigate muscle and bone loss while they are on the Martian surface.

The effects of Martian gravity on the human sensorimotor, cardiovascular, musculoskeletal, and immune systems, as well as effects on behavior, general health and performance, are unknown. The only data available have been obtained during short periods in parabolic flight. The plan is to use suspension systems (body inclination, suspension with springs or counterweight, lower body positive pressure) to assess how simulated short-duration (e.g., minutes) exposure to Martian gravity affects functional performance.

After they return from 6 months in space, ISS crewmembers will be exposed to head-up tilt (HUT) to investigate physiological adaption from 0 g to Martian gravity. Immediately after landing and a few days later, ISS crewmembers will be placed in a sitting or standing 22.3° HUT position. In this HUT position, the gravitational acceleration component along the long axis of the body is 0.38 g, which is equivalent to Martian gravity.^1^ We need to know whether crewmembers’ sensorimotor and cardiovascular systems will function adequately in the early post-landing phase on Mars and how long will it take for them to gain full functionality. Standard protocols currently used for assessing the recovery of physiological functions after spaceflight will be performed and the results will be compared with the database of responses to these protocols during normal recovery.

## Gap 3—AG duration

Because NASA is considering AG to counteract the effects of microgravity in humans, and this includes potentially rotating either the entire spacecraft or a device within a spacecraft, we must determine early in the vehicle design process what centrifugation force levels and rotation rates humans can adapt to. It is particularly important to determine as soon as possible whether there are any obvious showstoppers. Studies in the 1960s using slowly rotating rooms and large-radius centrifuges have provided theoretical limits for the rotation rates and radii to which humans can adapt.^35^ These limits are generally assumed to be correct, but they must be validated by experimental evidence. Recent work suggests that a human’s ability to adapt to rotating environments might be less limited than these earlier studies had anticipated.

Short-radius centrifugation has been used during 5–28 day bed rest studies to generate 1–2 g at the heart along the subjects’ longitudinal body axis (Gz) for periods ranging from 1–2 h per day. Intermittent centrifugation has been shown to attenuate orthostatic intolerance, and reduce alterations in parasympathetic activity, exercise capacity, and postural stability after bed rest. However, centrifugation did not prevent immune system deficiency, and the effects on bone loss were inconclusive, presumably because of the limited duration of bed rest.^36^ In addition, with the exception of two very short-duration (4–5 days) studies, the effects of multiple daily centrifugation sessions vs. a single bout of centrifugation have not been systematically studied in bed rest subjects. These studies suggest that repetitive, short-duration centrifugation sessions are more effective in mitigating orthostatic intolerance,^25^ neurovestibular symptoms,^37^ and neuroendocrine alterations^26^ than longer sessions.

In an upcoming study, 24 subjects will participate in a 60-day bed rest study at the:envihab facility in Germany. Subjects will be divided into three groups: one group of subjects will be exposed to bed rest alone; a second group will be exposed to 1 Gz at the center of mass (about 2 Gz at the feet) for a continuous period of 30 min per day (i.e., half of the current duration of physical exercise on board the ISS); a third group will be exposed to six bouts a day of the same centrifugation level for five minutes each session. Musculoskeletal deterioration, cerebral and cardiovascular changes, neurocognitive performance, and brain plasticity will be compared across these three groups of subjects before, during, and after the bed rest.

Dry immersion causes the same physiological changes as head-down bed rest, but changes occur after a relatively shorter duration of exposure. The effectiveness of intermittent Gz centrifugation using a short-radius centrifuge has been previously investigated in volunteers during dry immersion studies lasting 3–28 days.^38,39^ The duration of centrifugation ranged from 40–90 min per day, but only for a limited number of days. Russian investigators are currently performing dry immersion studies combined with daily intermittent centrifugation sessions.^23^

It can be argued that the ‘ideal’ AG level for humans might not be continuous 1 g, but gravity levels that are contingent on the duration and the amount of acceleration generated during walking in 1 g; therefore, a combination of both centrifugation and exercise might be a preferable countermeasure for the effects of microgravity. Studies are planned to address the biomechanics and constraints of performing acute exercise during rotation on a short-radius centrifuge.^40,41^ When adequate training protocols are established, bed rest studies will determine the effectiveness of AG exercises to counteract immobilization-induced deconditioning. ESA is planning two more bed rest studies to compare the mitigation effects of exercise regimes that will be performed outside the centrifuge and during centrifugation. ESA’s long-term plan also includes bed rest studies using intermittent lunar and Martian gravity (subjects in HUT at 9.5° or 22.3°, respectively) for a duration that is similar to the duration of planned planetary surface activities (HDT at −6° the rest of the time). Another issue that must be addressed is the optimal time of day when intermittent centrifugation is applied because animal studies have shown that exposure to altered gravity levels changed homeostatic parameters and circadian rhythms.^42^

## Gap 4—Health effects of AG

In previous centrifugation studies, the subjects’ heads were immobilized and the effects of cross-coupled angular and Coriolis accelerations during head and limb movements were not investigated. In addition, limited data are available on how gravity gradient during short-radius centrifugation affects physiological functions, and the exact etiology of anatomical ocular changes and visual functional changes observed during and after spaceflight is unknown at present. Short-radius centrifugation that generates Gz centrifugal force from head to foot might potentially mitigate this spaceflight associated neuro-ocular syndrome (SANS) by counteracting the headward fluid shift, reducing vascular and lymphatic congestion, and allowing outflow of cerebrospinal fluid.

**Fig. 3:**
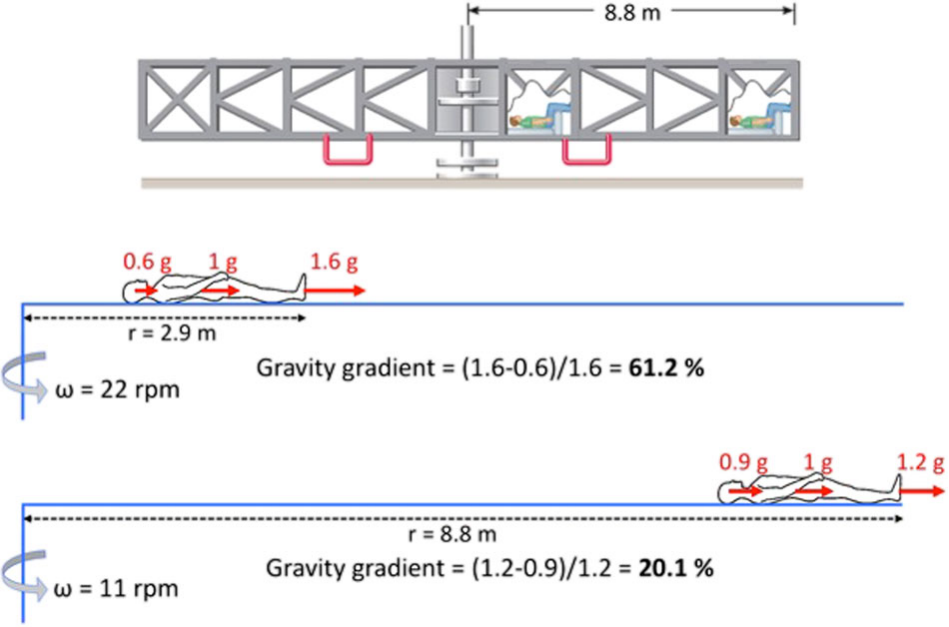
Design of an experiment using a large radius centrifuge to investigate the effects of gravity gradient. Top panel: centrifuge drawing (courtesy of NASA). Middle and lower panels: comparison between the amplitude of the +Gz centrifugal forces generated at the inner ear, center of mass, and feet in a supine subject placed close (*r* = 2.9 m) and far (*r* = 8.8 m) from the axis of rotation. ω: rotation rate. The formula for calculating the gravity gradient across the subject’s long body axis is shown for both conditions

Objects in a rotating environment have a different ‘weight’, depending on their distance from the center of rotation. This gravity gradient makes it difficult to move limbs and change body positions in a rotating room. The gravity gradient is also responsible for different AG levels at the head, heart, and feet in supine subjects who are on a short-radius centrifuge. These effects will be assessed by comparing the physiological responses and the effects of handling objects as the radius of centrifugation is increased. In a long-radius centrifuge the subject can be placed at various distances from the axis of rotation as shown in Fig. 3.

This ground-based effort is required to evaluate the effects of AG levels on ocular anatomy and function, head and neck vascular parameters, and intracranial pressure. A long-radius centrifuge is required in which subjects are exposed to Gz with a small gravity gradient. Human tolerance to + Gz is about 60 min at 4 g. This project will determine level of the gravity at the head that is sufficient to cause caudal drainage. The proposed project can also potentially study the effects of centrifugation on terrestrial populations with elevated intracranial pressure and vision issues of unknown etiology e.g., idiopathic intracranial hypertension or pseudo tumor cerebri, which share some of the signs and symptoms of SANS.

Slowly rotating rooms have been designed to study human behavioral and physiological responses to rotating environments, and studies focus on the effects of rotation rate and the resulting spatial disorientation generated by Coriolis and cross-coupled accelerations during head or body movements. A series of investigations were performed in Pensacola in the 1960s.^2,3^ After 12 days at 10 rpm, most subjects had not adapted completely and experienced apathy, slower reaction times, and lack of motivation. Progressively increasing the speed of rotation helped the subjects to adapt quicker.^43^ The various rotation rates (both incremental and discrete), and training techniques for adapting to the rotating environment must be tested, and must include more physiological endpoints than in the earlier studies. Precise measures of the subjects’ body movements were not obtained during the earlier slow rotating room studies. In fact, most subjects did not perform the prescribed tasks (presumably to avoid motion sickness) and remained inactive.^29^ Therefore, it is important to study how subjects walk, move, manipulate objects, and how they interact with other devices and control interfaces in slowly rotating rooms—tasks that are required to efficiently work in a rotating environment.^44^

A greater number of behavioral and physiological responses than those measured in the earlier slow rotating room studies need to be investigated. These responses include but are not limited to the evaluation of changes in the sensorimotor, cardiovascular, and musculoskeletal system; behavior, health and performance assessment; and eye and vision changes. In addition, we need to evaluate the consequences of long-duration exposure to rotating environment on cognition, functional performance, exercise, material handling, interaction with other devices and control interfaces, simulated extra-vehicular activity, and selected operational task performance.

## Gap 5—Validation studies

We do not know whether the AG prescription determined during ground-based studies will be effective, acceptable, and safe for the crew in space. Although ground-based studies have the potential for determining a sound AG prescription (including AG level and exposure duration/frequency), validation can only be performed in space. Given the time constraints of this project, it is most likely that a full validation using an ISS-based human rated centrifuge won’t be feasible.

Adaptation to a rotating environment is different on Earth than in space because gravity is perpendicular to the plane of rotation of the slow rotating room on Earth, whereas the centripetal acceleration vector is in the plane of rotation in space. In microgravity, the cross-coupled effect of a particular head movement with respect to the body is different for each direction the person faces. This effect could confound adaptation to the rotating environment in space and re-adaption to normal conditions after return from space. A large, very slow 1 g centrifuge where a subject can walk 45° relative to the plane of rotation will approximate the in-plane condition in space and help determine if adaptation is different. Alternatively, an aircraft that produces sustained coordinated 1 g level bank and turn can be used to simulate in-plane conditions. Two or three-hour exposure to these conditions may be sufficient to evaluate adaptation and subsequent recovery problems.

The rodent centrifugation studies on board the ISS mentioned above will provide opportunities to compare the effectiveness of the AG prescription in the ground-based and space conditions. The rodents will be exposed to centrifugation for 90 days to investigate the long-term effects of AG. Tests will be repeated during several times to fine-tune the AG prescription in orbit and evaluate inter-individual differences.

For humans, a simple, lightweight onboard centrifuge could be used to assess impacts of vibration level, motion sickness, or crew time during centrifugation inside a space vehicle. JAXA is considering implementing a lightweight, human-powered short-radius centrifuge inside its HTV-X transfer vehicle to be used as a test bed when the vehicle is docked with the ISS. The objectives of this engineering demonstration will be to assess the acceleration loads, g jitters, and the airflow, and to identify potential hazard and safety issues.

The Engineering Division at NASA Johnson Space Center is currently working on concepts for a 5-m radius human centrifuge inside the Deep Space Habitat (DSH) that will be compatible with NASA’s new Space Launch System. The first steps will be to perform studies using a ground model of the DSH centrifuge (based on the requirements defined above to validate the AG prescription: AG level, duration, frequency, etc.) as recommended by the outcomes of Gaps 1, 2, 3, and 4. The ground-based AG prescription would then be validated using the DSH centrifuge in space. The main objective is to get a quick feedback on whether the AG dose determined from ground-based studies (and fine-tuned based on the results of the comparison between animals ground-based and flight studies) is effective. This flight project will also investigate a number of factors associated with crew compliance, crew safety, and operational issues during cis-lunar exploration missions.

## Conclusion

The international AG roadmap includes both ground-based and space-based projects. Studies involve human subjects, as well as animal and cell models. The availability of human subjects and centrifuges for carrying out studies in space are limited. In addition, animal models allow controlling factors such as diet, fluid intake, physical exercise, sleep, stress, and genetic background.^21,45^

The international AG roadmap is not only translational across species. It is also translational across systems. AG is an integrative approach that included biological, physiological, behavioral, and human factor aspects. It is also a multidisciplinary effort for space physiologists, crew surgeons, astronauts, vehicle designers, and mission planners to review, evaluate, and discuss the issues for incorporating AG technologies into the vehicle design. Commitments to implement AG can only happen after acceptance of a well-argued requirement from the aerospace medicine community.

It is expected that the international AG roadmap will standardize study protocols, and measurements across studies, thus facilitating comparison of results. For example, for studies of intermittent centrifugation during bed rest and dry immersion, the roadmap suggests (a) keeping the rotation rate constant across studies; (b) considering protocols where the AG prescription would be individualized; and (c) adding measurements such as subjective comfort, operational performance, cognition, and metabolomics. The AG roadmap will also be useful for identifying specific barriers that are likely to be encountered during the various phases of the research plan. It is expected that representatives of space agencies will review the AG roadmap on an annual basis to adjust or revise the content and timing of research tasks based on changes in their strategic plans. Thus, decision makers using the roadmap will be more likely to anticipate the effort needed to implement AG in manned space mission within budget and on time.
